# Association Between Levels of Magnesium and Diabetic Retinopathy in Diabetic Patients with Type 2 Diabetes Mellitus: An Updated Systematic Review and Meta-Analysis

**DOI:** 10.3390/nu18071162

**Published:** 2026-04-06

**Authors:** Eman A. Kubbara, Sara Z. Hamdan, Tassneem Awad Hajali, Mohamad Y. Rezk, Hamdan Z. Hamdan

**Affiliations:** 1 Clinical Biochemistry Department, Faculty of Medicine, Rabigh Branch, King Abdulaziz University, Rabigh 21911, Saudi Arabia; eadm@kau.edu.sa; 2 Department of Medicine, University Hospital Kerry, Rathass, V92NX94 Tralee, County Kerry, Ireland; 3 Department of Clinical Science, College of Medicine, Sulaiman Al Rajhi University, P.O. Box 777, Al Bukayriah 51941, Saudi Arabia; 4 Department of Physiology, College of Medicine, Qassim University, P.O. Box 6655, Buraidah 51452, Saudi Arabia; 5 Department of Pathology, College of Medicine, Qassim University, P.O. Box 6655, Buraidah 51452, Saudi Arabia

**Keywords:** diabetic retinopathy, proliferative diabetic retinopathy, non-proliferative diabetic retinopathy, magnesium levels, diabetes mellitus type 2

## Abstract

Background/Objectives: Magnesium is an intracellular cation that plays important roles in metabolism and insulin signaling. The evidence of association between magnesium levels and diabetic retinopathy is limited by small study effects. Therefore, this systematic review and meta-analysis aim to update the current evidence. Methods: A comprehensive search of PubMed, ScienceDirect, Google Scholar, and Scopus was conducted from database inception to January 2026 to identify studies examining magnesium levels and diabetic retinopathy. The pooled standardized mean difference in magnesium levels between type 2 diabetic patients with retinopathy and those without retinopathy was estimated using the “meta” package in R software. Results: We included seventeen studies which assessed magnesium levels in 1100 patients with diabetic retinopathy and 1132 diabetic controls without retinopathy. The random-effects model indicated significantly lower magnesium levels in patients with diabetic retinopathy compared to diabetic controls [SMD = −1.19, 95% CI (−1.68; −0.70); *p* < 0.0001; I^2^ = 95%]. Sensitivity analysis retained all studies, and no evidence of publication bias was detected. Subgroup analyses demonstrated consistent findings across geographic regions (Asian versus non-Asian), study designs (case–control versus cross-sectional), and magnesium assay methods except enzymatic method. Meta-regression analysis revealed that year of publication (coefficient = 0.061; *p* = 0.009) and non-Asian studies (coefficient = 2.376; *p* = 0.001) were positively associated with the pooled effect size, while the NOS quality score was inversely associated (coefficient = −0.709; *p* = 0.035). The magnesium levels were significantly lower in patients with proliferative diabetic retinopathy compared with those with non-proliferative diabetic retinopathy using a fixed effect model [SMD = −1.41, (95% CI: −1.83; −1.00); *p* < 0.01; I^2^ = 32%; Cochran’s Q statistic (Q = 1.46, *p* < 0.23)]. The certainty of the generated evidence is rated as low certainty. Conclusions: This systematic review and meta-analysis conclude that magnesium levels are significantly lower in patients with diabetic retinopathy than in diabetic controls without retinopathy. A potential association between hypomagnesemia and the development of diabetic retinopathy in individuals with type 2 diabetes is suggested; therefore, the clinician may check and adjust magnesium levels in patients with type 2 diabetes mellitus.

## 1. Introduction

Diabetes mellitus remained a global health concern, with its prevalence reaching 830 million cases worldwide in 2022, according to the WHO [1]. The majority of these reported cases had type 2 diabetes mellitus. Diabetic retinopathy is among the most common microvascular complications of diabetes [2,3] and is one of the leading causes of preventable blindness [4]. It affects around 80% of diabetic patients [5]. Diabetic retinopathy is multifactorial and thought to involve oxidative stress, endothelial dysfunction, inflammation, and chronic hyperglycemia [6,7,8].

Magnesium is a divalent cation that is needed as a cofactor in over 300 enzyme-catalyzed reactions, including those involved in glucose metabolism, ATP-dependent processes, insulin signaling, and vascular tone regulation [9]. Disturbance in magnesium levels has been associated with insulin resistance, endothelial dysfunction, and increased oxidative stress in the background of type 2 diabetes mellitus [10,11,12]. These processes are recognized as central mechanisms underlying the development of diabetic microvascular complications, including retinopathy.

In ocular tissues, magnesium is highly distributed inside the retina photoreceptor and lens. It is localized within the retinal cytosol as well as different subcellular organelles including the mitochondria [13]. Magnesium is mainly needed to generate ATP inside the retina, and this energy supports membrane transport systems such as Na^+^-K^+^-ATPase and Ca^2+^-ATPase, which maintain ionic balance and preserve retinal homoeostasis [14,15]. Additionally, magnesium plays a neuroprotective role in retinal neurons by suppressing the exaggerated activity of the inducible nitric oxide synthase (iNOS) enzyme, thereby limiting nitrosative stress [16]. Moreover, magnesium also acts as a natural blocker for *N*-methyl-d-aspartate (NMDA) receptors as well as calcium channels in retinal neurons [14]. Therefore, reduced magnesium levels may permit excessive intracellular calcium and sodium influx, leading to excitotoxic neuronal damage that contributes to diabetic retinopathy [17,18,19].

Several observational studies have reported an association between reduced magnesium levels and diabetic retinopathy [20,21], although some studies failed to demonstrate this association [22]. A previous meta-analysis reported an association between magnesium levels and diabetic retinopathy [23]; however, the available evidence was derived from a limited number of studies, characterized by substantial heterogeneity and relatively small effect sizes. In addition, several relevant studies have been published subsequently [24,25,26], therefore the current meta-analysis was conducted to update the current synthesized evidence about magnesium levels and diabetic retinopathy. Clinically, magnesium is a modifiable factor that is widely available, easily assessed at clinics, and inexpensive. These features qualify magnesium as a potential useful biomarker for risk stratification and early identification of diabetic patients at higher risk of retinopathy progression, which may contribute to the prevention of disease progression and avoidance of vision loss. Considering the likely impact of magnesium status on the health outcomes of patients with diabetes [27,28], generating updated, robust evidence is of considerable importance to clinicians, healthcare providers, policymakers, and biomedical researchers. Therefore, the present study was conducted to update the current evidence regarding the association between magnesium levels and diabetic retinopathy.

## 2. Materials and Methods

**Study protocol, registration and search strategy**

This systematic review and meta-analysis followed the Preferred Reporting Items for Systematic Reviews and Meta-Analyses (PRISMA) guidelines, as outlined in the PRISMA checklist (Table S1). The full protocol has been registered in PROSPERO registry under the registration number CRD420261321335. Eligible studies were those that assessed magnesium levels in patients with type 2 diabetes mellitus complicated by diabetic retinopathy and compared them with levels observed in diabetic patients without retinopathy. A comprehensive literature search was conducted across PubMed, ScienceDirect, Google Scholar, and Scopus to identify relevant studies from inception until January 2026. The search strategy combined MeSH and non-MeSH terms using the Boolean operators [AND], [OR] and [NOT] to optimize search results. The complete list of search terms and detailed search strategy is provided in Table S2.

The search framework was developed according to the Population, Intervention, Comparison, Outcome, and Study design (PICOS) strategy:

- P (Population): type 2 diabetes mellitus OR insulin independent diabetes mellitus OR IIDM OR NIDDM
- I (Intervention): magnesium OR Mg
- C (Comparison): type 2 diabetic patients without diabetic retinopathy
- O (Outcome): diabetic retinopathy OR proliferative diabetic retinopathy OR non-proliferative diabetic retinopathy
- S (Study design): Case–control OR cross-sectional OR cohort

Three investigators (MYR, TA, and EAK) independently performed the literature search and initial screening of potentially eligible studies. Titles and abstracts were evaluated to determine eligibility for full-text reviews. The reference lists of included articles were also manually screened to identify further relevant studies. Disagreements between reviewers were resolved through discussion, with arbitration by a senior reviewer (HZH).

**Inclusion criteria**

Studies were considered eligible if they: assessed serum or plasma magnesium levels in patients with type 2 diabetes mellitus with diabetic retinopathy and compared them with diabetic patients without retinopathy; reported magnesium concentrations as mean with standard deviation (SD) or provided data that could be converted to mean (SD); articles designed as case–control, cohort, or cross-sectional study; and published in the English language.

**Exclusion criteria**

Studies were excluded if they: compared magnesium levels between diabetic patients and non-diabetic controls; reported magnesium levels in formats not convertible to mean (SD); included patients receiving magnesium supplementation; were published as letters to the editor, conference abstracts, case reports, review articles, or animal studies; or were published in languages other than English.

**Definition of the outcome of the interest**

The primary outcome of this meta-analysis was to determine the association between magnesium levels and diabetic retinopathy in patients with type 2 diabetes mellitus. This association was assessed by calculating the standardized mean difference (SMD) of magnesium levels between diabetic patients with retinopathy and those without retinopathy. Diabetic retinopathy was defined according to the American Academy of Ophthalmology criteria [29] and classified into non-proliferative diabetic retinopathy (NPDR) and proliferative diabetic retinopathy (PDR).

**Assessment of risk of bias**

The methodological quality of the included studies was evaluated using the Newcastle–Ottawa Scale (NOS). The standard NOS was applied to case–control and cohort studies, whereas a modified version was used for cross-sectional studies. The scale evaluates three domains: selection of participants; comparability of study groups; and outcome assessment. Studies were rated using a star system with a maximum score of nine stars. Studies scoring seven or more stars were classified as high quality, those scoring three to six stars were considered moderate quality, and studies scoring fewer than three stars were categorized as low quality.

**Data extraction**

Data extraction was performed independently by three reviewers (TAH, EAK, and MYR) after the eligible studies were retrieved. The following variables were extracted: first author’s name, publication year, country, magnesium levels in diabetic patients with and without retinopathy, laboratory method used for magnesium determination, study design, mean (SD) age, and sample size. All extracted data were recorded in a Microsoft Excel spreadsheet and verified prior to statistical analysis. When magnesium levels were reported in formats other than mean (SD), the corresponding mean and SD were estimated using established statistical methods [30].

**Evidence certainty using the GRADEpro GDT tool**

The certainty of the evidence was assessed using the GRADEpro GDT online platform [31]. This scheme assesses the quality of evidence across the domains of risk of bias, inconsistency, indirectness, and imprecision.

**Statistical analysis**

All statistical analyses were carried out using R software version 4.5.0 (R Foundation for Statistical Computing, Vienna, Austria), as previously described [32,33]. To compare magnesium levels between diabetic retinopathy cases and diabetic controls, the pooled standardized mean difference (SMD) was estimated using the meta package (version 7.0) via the metacont function. Inter-study heterogeneity was assessed using Cochran’s Q test and Higgins’ inconsistency index (I^2^). Significant heterogeneity was defined as *p* < 0.01 for the Q test and I^2^ > 50%. When heterogeneity was present, a random-effects model was applied; otherwise, a fixed-effects model was used. Pre-specified subgroup analyses were conducted based on: Geographic region, Study design, and Magnesium measurement method. Meta-regression analysis was performed to examine the influence of potential covariates on pooled SMD estimates. Sensitivity analyses were conducted to assess the robustness of the pooled results by examining the influence of individual studies. Publication bias was assessed visually using funnel plots and quantitively with Egger’s regression test. A cumulative meta-analysis was performed using the metacum function in the meta package as described [34]. Statistical significance level was set at *p* < 0.05.

## 3. Results

**Study selection**

**Figure 1:**
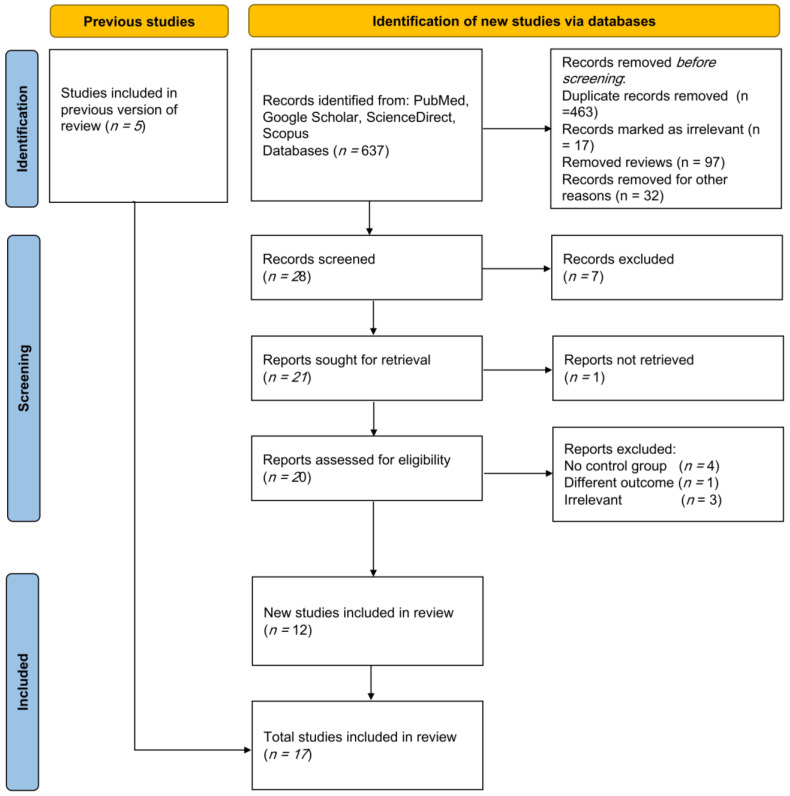
Study flowchart showing studies identification, screening, and inclusion process.

The comprehensive search across the selected databases identified 637 records. After removing duplicate entries and excluding case reports, animal studies, editorials, conference proceedings, and review articles, 28 records remained eligible for screening. Titles and abstracts were then carefully examined, resulting in 21 studies being considered for full-text review. Following full-text assessment, eight studies were excluded due to the absence of an appropriate control group, assessment of outcomes unrelated to the study objective, or lack of relevance. Ultimately, 12 newly identified studies, together with five studies retrieved from the previous review, fulfilled the eligibility criteria and were included in the final meta-analysis (Figure 1).

**Features of selected studies**

A total of seventeen studies were included in the meta-analysis, all of which investigated magnesium levels in patients with type 2 diabetes mellitus with and without diabetic retinopathy. Overall, the included studies comprised 1100 patients with diabetic retinopathy and 1132 diabetic controls without retinopathy. Two studies compared the levels of magnesium in proliferative diabetic retinopathy with cases with non-proliferative diabetic retinopathy [35,36].

**Figure 2:**
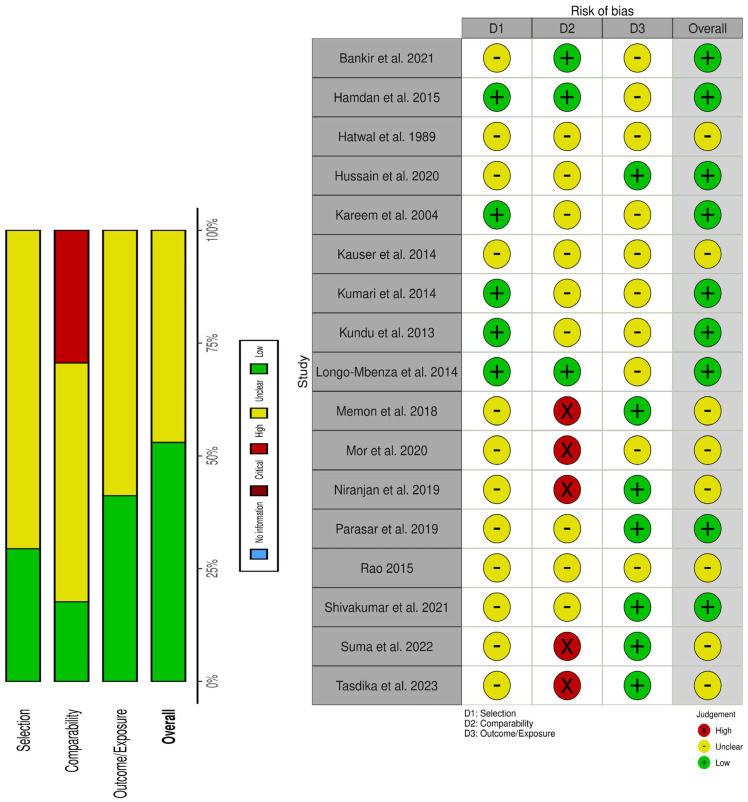
Risk of bias assessment graph and summary [18,20,21,24,25,26,35,36,37,38,39,40,41,42,43,44,45].

Geographically, fourteen studies were conducted in Asia, with eleven studies in India [24,25,35,36,37,38,39,40,41,42,43], one in Pakistan [44], one in Bangladesh [26], and one in Iraq [21]. Two studies were conducted in Africa, one in Sudan [45] and one in the Democratic Republic of the Congo [18]. Only one study was conducted in Europe (Turkey) [20]. Regarding study design, seven studies employed a cross-sectional design [20,21,24,25,36,40,42], whereas ten studies used a case–control design [18,26,35,37,38,39,41,43,44,45]. In terms of magnesium measurement methods, nine studies used colorimetric methods [20,21,24,25,36,37,39,40,41], five used atomic absorption spectrometry [18,26,35,37,38,39,41,43,44,45], and another three studies used enzymatic methods [38,42,43]. According to the Newcastle–Ottawa Scale, nine studies were rated as high quality [18,20,21,24,38,39,41,42,45], whereas the remaining studies were considered moderate quality [25,26,35,36,37,40,43,44]; detailed quality assessments are presented in Table 1 and Figure 2.

**Magnesium levels meta-analysis results**

**Figure 3:**
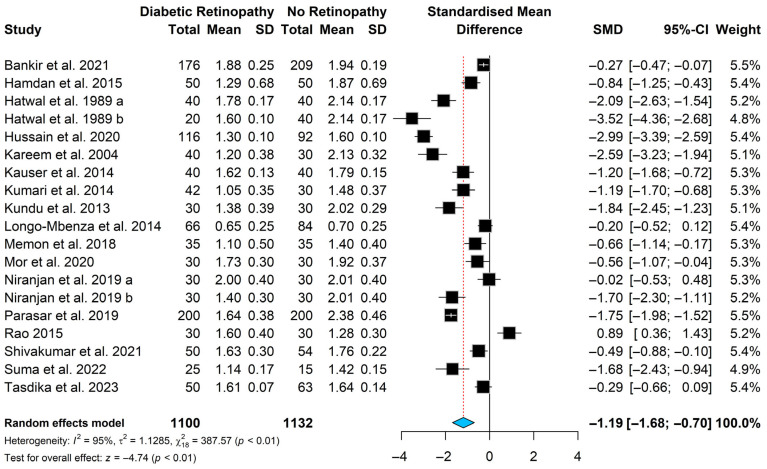
Forest plot for the magnesium levels meta-analysis in patients with diabetic retinopathy [18,20,21,24,25,26,35,36,37,38,39,40,41,42,43,44,45]. a includes NPDR. b includes PDR.

**Figure 4:**
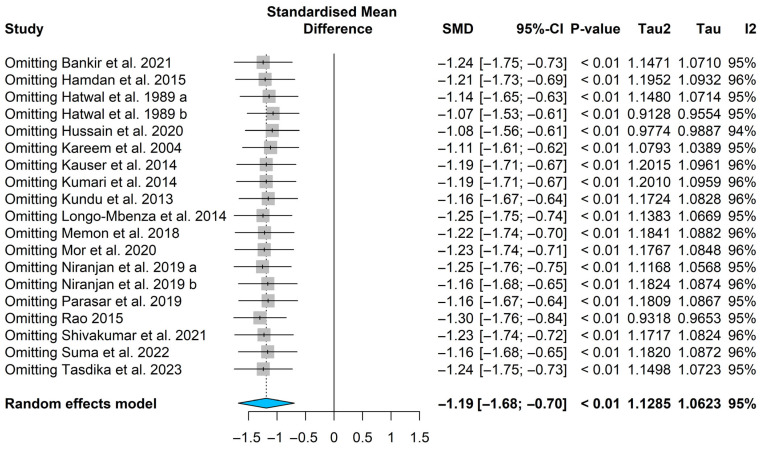
Magnesium levels meta-analysis in patients with diabetic retinopathy [18,20,21,24,25,26,35,36,37,38,39,40,41,42,43,44,45]. a includes NPDR. b includes PDR.

The pooled standardized mean difference (SMD) indicated that magnesium levels were significantly lower in patients with diabetic retinopathy compared with diabetic patients without retinopathy [SMD = −1.19, (95% CI: −1.68; −0.70); *p* < 0.0001], see Figure 3. Substantial heterogeneity was observed among the included studies, as indicated by Higgins’ inconsistency index (I^2^ = 95%) and Cochran’s Q statistic (Q = 387.57, *p* < 0.0001). Consequently, a random-effects model was applied. To investigate potential sources of heterogeneity, a sensitivity analysis was performed to determine whether any individual study exerted a disproportionate influence on the pooled estimate. In addition, a Baujat plot was generated to visually assess each study’s contribution to overall heterogeneity. Both the Baujat plot (Figure S1) and the sensitivity analysis indicated that no single study contributed disproportionately to the observed heterogeneity (Figure 4). Therefore, no studies were excluded from the final meta-analysis.

**Figure 5:**
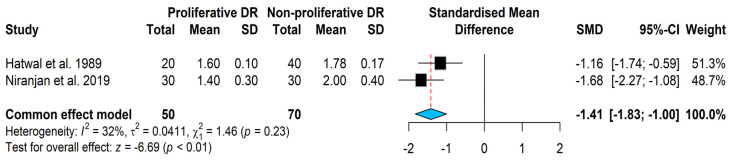
Magnesium levels meta-analysis in patients with proliferative diabetic retinopathy and non-proliferative diabetic retinopathy [35,36].

When comparing the magnesium levels in cases with PDR to cases with NPDR magnesium, levels were found significantly lower in cases with PDR using the fixed effect model [SMD = −1.41, (95% CI: −1.83; −1.00); *p* < 0.01; I^2^ = 32%; Cochran’s Q statistic (Q = 1.46, *p* < 0.23)], see Figure 5.

**Subgroups and Meta-regression analysis**

Subgroup analyses were performed to identify possible sources of heterogeneity. First, studies were stratified according to geographical region, categorized as Asian countries and non-Asian countries. The results showed significantly lower magnesium levels among patients with diabetic retinopathy in both groups. The Asian subgroup showed a pooled effect size of [SMD = −1.34 (95% CI: −1.90; −0.78); *p* < 0.01; I^2^ = 95.0%], while the non-Asian subgroup showed [SMD = −0.41 (95% CI: −0.77; −0.05); *p* < 0.01; I^2^ = 71.0%]. Both groups indicate that the overall effect was consistent across geographical regions, as seen in Table 2; Figure S2.

A similar pattern was observed in subgroup analyses based on study design. Studies were categorized as either cross-sectional or case–control. The cross-sectional subgroup demonstrated a pooled effect size of [SMD = −1.26 (95% CI: −1.94; −0.57; *p* < 0.01; I^2^ = 97.0%], whereas the case–control subgroup showed [SMD = −1.14 (95% CI: −1.86; −0.42; *p* < 0.01; I^2^ = 94.0%]. In both subgroups, magnesium levels were significantly lower in patients with diabetic retinopathy compared with diabetic controls, as shown in Table 2; Figure S3.

Additional subgroup analyses were conducted according to the laboratory method used for magnesium determination, including colorimetric methods, enzymatic methods, and atomic absorption spectrometry. Studies using colorimetric methods reported significantly lower magnesium levels among cases [SMD = −1.32; (95% CI: −1.95; −0.69); *p* < 0.01; I^2^ = 96.0%]. Similarly, studies using atomic absorption spectrometry also demonstrated significantly reduced magnesium levels [SMD = −1.23; (95% CI: −2.23; −0.23); *p* = 0.02; I^2^ = 94.0%]. In contrast, studies using enzymatic methods did not show a statistically significant difference between cases and controls [SMD = −0.69; (95% CI: −2.27; 0.88); *p* = 0.38; I^2^ = 98.0%], see Table 2 and Figure S4.

Meta-regression analysis was further performed to investigate the potential influence of study-level covariates on the pooled effect size. The model included continuous variables (NOS score, year of publication, and study sample size) as well as categorical variables (method of magnesium assay and study continent). The analysis showed that year of publication (coefficient = 0.061; *p* = 0.009) and non-Asian geographic location (coefficient = 2.376; *p* = 0.001) were positively associated with magnesium levels. In contrast, the NOS quality score demonstrated a significant inverse association with magnesium levels (coefficient = −0.709; *p* = 0.035), see Table 3.

**Publication bias**

**Figure 6:**
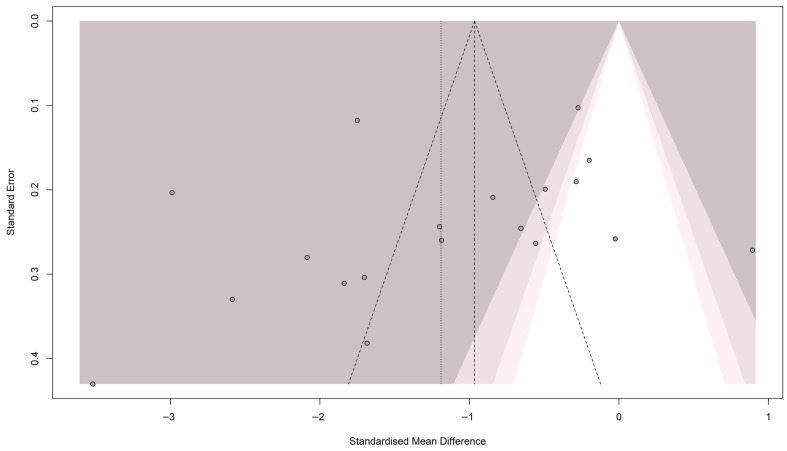
Funnel plot for pooled studies in magnesium levels meta-analysis in patients with diabetic retinopathy [18,20,21,24,25,26,35,36,37,38,39,40,41,42,43,44,45].

Visual inspection of the funnel plot showed a symmetrical distribution of studies, suggesting no significant publication bias, as shown in Figure 6. This observation was supported by Egger’s regression test, which showed no statistical significance (t = −1.25; *p* = 0.233).

**Cumulative meta-analysis of magnesium levels**

**Figure 7:**
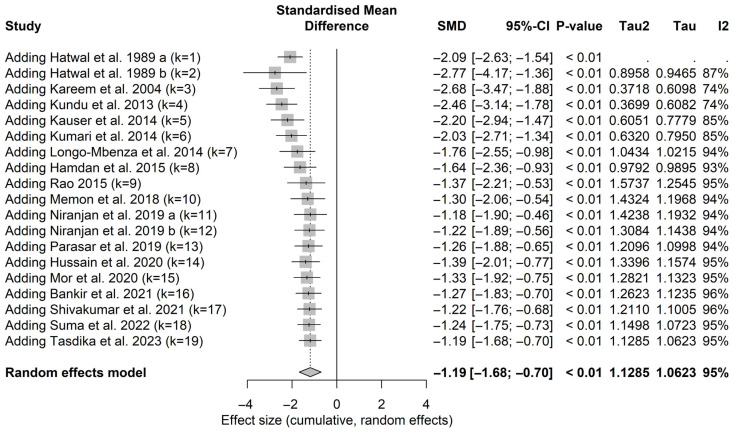
Cumulative meta-analysis for pooled studies in magnesium levels meta-analysis in patients with diabetic retinopathy [18,20,21,24,25,26,35,36,37,38,39,40,41,42,43,44,45]. a includes NPDR. b includes PDR.

Cumulative meta-analysis revealed that the overall pooled standardized mean difference remained consistently significant as additional studies were included over time. The confidence intervals did not cross the null effect after the earliest included study and became progressively narrower with the addition of subsequent studies. This evidence suggests that the precision and robustness of the pooled estimate improved over time, particularly with the inclusion of the most recent studies, see Figure 7.

**GRADE certainty of evidence results**

According to the GRADE approach, the certainty of evidence for the outcome “magnesium levels (SMD) in diabetic retinopathy” was judged to be low. This assessment was attributed to a serious risk of bias, arising from observational study designs, and a very serious inconsistency, indicating the substantial heterogeneity seen across studies, see Table 4.

## 4. Discussion

The current systematic review and meta-analysis update the evidence on the association between magnesium levels and diabetic retinopathy in patients with type 2 diabetes mellitus. The principal finding of this study is that patients with diabetic retinopathy exhibit significantly lower circulating magnesium levels compared with diabetic patients without retinopathy. Our results are in line with the previous meta-analysis by Adiwinoto et al. 2021 [23]. However, the earlier meta-analysis included only 9 studies comprising 411 cases and 403 controls [23], whereas the current study includes 17 studies comprising 1100 patients with diabetic retinopathy and 1132 diabetic controls without retinopathy. Moreover, the present study extends prior work by conducting meta-regression analysis in addition to subgroup analysis, enabling the identification of covariates influencing the overall effect estimate, wheareas the latest meta-analysis did not perform meta-regression and had a small effect size [23]. We also conducted a cumulative meta-analysis to examine the chronological evolution of the evidence and assessed the certainty of evidence using a formal grading approach. These methodological refinements provide a more comprehensive and robust synthesis of the available data on the association between magnesium levels and diabetic retinopathy.

In the current study, magnesium levels remain significantly lower in cases with diabetic retinopathy across subgroup analyses stratified by geographic region and study design. The association was consistent in studies conducted in Asian and non-Asian countries and in both cross-sectional and case–control designs. Although statistical heterogeneity remained high across subgroups, the persistence of the effect suggests that the observed association is unlikely to be affected by geographic region or study design differences and is more likely a true biological effect. Additionally, subgroup analysis according to magnesium assay methods revealed that studies employing colorimetric and atomic absorption techniques consistently demonstrated significantly lower magnesium levels in cases compared to controls. Likewise, studies employing enzymatic methods followed a similar direction, but the result was not statistically significant. The enzymatic hexokinase method was used in the included studies. When compared with atomic absorption spectrometry, the hexokinase assay showed superior accuracy and precision for estimating clinical magnesium concentration [18]. However, as with other enzymatic reactions, hexokinase activity is sensitive to variations in reaction conditions such as pH and temperature [18], which may vary across laboratory settings due to different calibration settings. Therefore, this variability could be a potential source of heterogeneity.

It is worth to be mentioning that magnesium levels reported across the included studies ranged between 1.1 and 2.3 mg/dL, except for the study by Longo-Mbenza et al. 2014 [18], who reported lower values ranging from 0.65 to 0.7 mg/dL. In Longo-Mbenza et al. study, magnesium concentrations were measured using atomic absorption technique. Although atomic absorption is considered technically sound [46], it needs careful optimizations of analytical conditions, including background correction and matrix optimization to reduce chemical interference with magnesium [47]. Perhaps inadequate optimization may contribute to reduced levels of magnesium observed in Longo-Mbenza et al. study.

In this study, magnesium levels were significantly lower in patients with PDR compared with those with NPDR. This finding is in line with the previous meta-analysis evidence [23] and with the study by Niranjan et al. [36], which reported that the lowest magnesium levels were observed in patients with PDR, accompanied by the highest concentrations of endothelin-1 (ET-1) and vascular endothelial growth factor-A (VEGF-A) compared with NPDR patients. Notably, ET-1 has been associated with oxidative stress and increased expression of proinflammatory cytokines such as TNF-alpha and IL-6 [48,49,50]. On the other hand, VEGF-A is a known marker of retinal ischemia severity and a major inducer of neovascularization in diabetic retinopathy [48,51]. Interestingly, low magnesium levels have been linked to increased oxidative stress and endothelial dysfunction [7,52]. Taken together, perhaps the relationship between magnesium levels and diabetic retinopathy severity may follow a dose–response pattern.

Another finding in this study is that meta-regression analysis showed that higher Newcastle–Ottawa Scale scores were inversely associated with magnesium levels, whereas more recent publication year and studies conducted in non-Asian countries were directly associated with magnesium levels. Although there is no clear mechanistic explanation for these findings, environmental and dietary factors may contribute. For instance, soil analyses from agricultural regions in eastern Sudan have reported relatively high magnesium content [53]. Also, in Turkey, the dietary intake of type 2 diabetic patients showed that only 23.5% had inadequate magnesium intake [54]. Nevertheless, dietary magnesium intake was not assessed in most included studies, limiting interpretation. Other important covariates known to be determinants of diabetic complications, including diabetic retinopathy, such as glycemic control, diabetes duration, and age, may partially explain the observed heterogeneity. However, these variables were inconsistently reported across the included studies.

Magnesium is an intracellular cation and plays essential physiological and metabolic roles [55]. It contributes to maintaining the membrane potential, regulates intracellular ion fluxes, and acts as a cofactor in numerous enzymatic reactions involved in carbohydrate and lipid metabolism [56]. Magnesium is also required for insulin secretion, insulin receptor binding, and post-receptor intracellular signaling [57]. These functions provide a mechanistic rationale for the development of insulin resistance in the background of low magnesium levels. Indeed, hypomagnesemia has been consistently associated with poor glycemic control, which is a central determinant of microvascular complications, including diabetic retinopathy [57]. Beyond its role in insulin signaling [58], low magnesium has been linked to increased mitochondrial production of reactive oxygen species and heightened oxidative stress [52]. Oxidative stress is a well-established contributor to retinal endothelial dysfunction, pericyte loss, and capillary basement membrane thickening in diabetic retinopathy [7,52]. The causes of low magnesium levels in diabetic patients may result from inadequate dietary intake or increased urinary magnesium loss, both of which are common in diabetes [59,60]. Clinical trials investigating magnesium supplementation in diabetic populations have demonstrated beneficial outcomes in terms of lowering oxidative stress markers, improving insulin resistance indices, lowering fasting glucose, and glycated hemoglobin levels [27,28]. Collectively, these beneficial effects of magnesium support the view that magnesium is a potentially modifiable factor for diabetic retinopathy. However, to date, no clinical trial has directly evaluated magnesium supplementation for the prevention or progression of diabetic retinopathy.

Regarding effect size interpretation, our pooled standardized mean difference of −1.19 corresponds to a large effect according to Hedges’ criteria (small <0.4; moderate 0.4–0.7; large >0.7) [61]. To enhance clinical interpretability, we converted the SMD into an absolute mean difference using a representative standard deviation (SD = 0.25 mg/dL) derived from a population-based diabetic cohort [19]. The estimated absolute mean difference is approximately 0.3 mg/dL. This magnitude of difference is clinically meaningful and comparable to those reported for other diabetic complications, including diabetic nephropathy [62], and diabetic foot ulcer [63]. Furthermore, Lu et al. reported that each 0.243 mg/dL increase in serum magnesium was associated with a 20% reduction in the risk of diabetic retinopathy [64], underpinning the potential clinical relevance of the observed deficit. To date, no published clinical trial has investigated the effect of magnesium supplementation on the progression or prevention of diabetic retinopathy. However, several meta-analyses on magnesium supplementation trials confirmed the beneficial effects of magnesium on oxidative stress and inflammatory biomarkers [65,66]. Moreover, another clinical trial meta-analysis showed a dose–response effect of magnesium supplementation on the reduction in both fasting blood glucose and glycated hemoglobin [56]. Altogether, these premises indicate a beneficial effect of magnesium supplementation on improving glycemic control, intracellular stress levels, and modulating inflammation. Hence, we can deduce that magnesium can, indirectly, slow or prevent the progression of diabetic retinopathy.

Although heterogeneity remained substantial, the pooled results were robust in sensitivity analyses, and no evidence of publication bias was detected. The GRADE assessment indicated low certainty of evidence, primarily due to high heterogeneity and the observational nature of included studies. Notably, cumulative meta-analysis demonstrated progressive narrowing of confidence intervals with the addition of each study, reaching the narrowest interval after the inclusion of the most recent study by Tasdika et al. [26] suggesting that the overall estimate is not driven by a small number of studies.

The current meta-analysis updates and strengthens the synthesized evidence regarding the association between reduced magnesium levels and diabetic retinopathy in T2DM. Nevertheless, several limitations should be addressed to improve the readability and interpretation of the findings. First, dietary magnesium intake was not assessed in most studies. Second, renal function and urinary magnesium excretion were not measured in the included studies. Third, all included studies were observational (case–control or cross-sectional), precluding causal inference. Finally, temporal relationships cannot be excluded. On the other hand, our study has numerous strong points. We systematically searched several databases and included 17 studies from seven countries, comprising 1100 cases and 1132 controls. All included studies were of high or moderate quality. Sensitivity analysis did not materially alter the pooled estimate, and no publication bias was detected. Subgroup analyses consistently demonstrated the same pattern of association.

## 5. Conclusions

The current systematic review and meta-analysis demonstrate a significant association between decreased magnesium levels and diabetic retinopathy in patients with type 2 diabetes mellitus. Primary care physicians, ophthalmologists, and endocrinologists are urged to consider assessing and correcting magnesium levels in patients with diabetes. Randomized controlled trials are needed to determine the impact of magnesium supplementation on the prevention or slowing the progression of diabetic retinopathy. Further studies with a longitudinal design are needed to establish causality.

## Figures and Tables

**Table 1:** Characteristics of the studies included in the meta-analysis of magnesium levels in diabetic retinopathy.

Included Studies	Country	Study Design	Sample Size Cases: Controls		Mg Levels mg/dL in Cases Mean (SD)	Mg Levels mg/dL in Controls Mean (SD)	Mg Assay	Cases Age Mean (SD)	Controls Age Mean (SD)	NOS
Bankir et al. 2021 [20]	Turkey	Cross-sectional	176	209	1.88 (0.25)	1.94 (0.19)	Calorimetric	53.7 (7.5)	54.1 (10.9)	7
Hamdan et al. 2015 [45]	Sudan	Case–control	50	50	1.29 (0.679)	1.87 (0.686)	Atomic absorption	59.0 (11.2)	56.0 (10.5)	8
Hatwal et al. 1989 a [35]	India	Case–control	40	40	1.781 (0.17)	2.139 (0.17)	Atomic absorption	N.A.	N.A.	6
Hatwal et al. 1989 b [35]	India	Case–control	20	40	1.604 (0.097)	2.139 (0.17)	Atomic absorption	N.A.	N.A.	6
Hussein et al. 2020 [21]	Iraq	Cross-sectional	116	92	1.3 (0.1)	1.6 (0.1)	Calorimetric	61.1 (9.8)	46.1 (10.2)	7
Kareem et al. 2004 [39]	India	Case–control	40	30	1.2 (0.38)	2.13 (0.32)	Calorimetric	N.A.	N.A.	7
Kauser et al. 2014 [40]	India	Cross-sectional	40	40	1.62 (0.13)	1.79 (0.15)	Calorimetric	53.13 (6.42)	52.77 (7.63)	6
Kumari et al. 2014 [38]	India	Case–control	42	30	1.05 (0.35)	1.48 (0.37)	Enzymatic method	64.07 (6.23)	56.30 (6.29)	7
Kundu et al. 2013 [41]	India	Case–control	30	30	1.38 (0.39)	2.02 (0.29)	Atomic absorption	N.A.	N.A.	7
Longo-Mbenza et al. 2014 [18]	Congo	Case–control	66	84	0.65 (0.25)	0.7 (0.25)	Atomic absorption	53.4 (13.6)	56.6 (12.4)	8
Memon et al. 2018 [44]	Pakistan	Case–control	35	35	1.1 (0.5)	1.4 (0.4)	Calorimetric	N.A.	N.A.	6
Mor et al. 2020 [37]	India	Case–control	30	30	1.73 (0.3)	1.92 (0.37)	Calorimetric	N.A.	N.A.	5
Niranjan et al. 2019 a [36]	India	Cross-sectional	30	30	2 (0.4)	2.01 (0.4)	Calorimetric	55.2 (8.3)	54 (8.5)	6
Niranjan et al. 2019 b [36]	India	Cross-sectional	30	30	1.4 (0.3)	2.01 (0.4)	Calorimetric	59 (7.2)	54 (8.5)	6
Parasar et al. 2019 [42]	India	Cross-sectional	200	200	1.64 (0.38)	2.38 (0.46)	Enzymatic method	50.86 (7.48)	N.A.	7
Rao 2015 [43]	India	Case–control	30	30	1.6 (0.4)	1.28 (0.3)	Enzymatic method	N.A.	N.A.	6
Shivakumar et al. 2021 [24]	India	Cross-sectional	50	54	1.63 (0.3)	1.76 (0.22)	Calorimetric	57.38 (8.53)	61.15 (8.88)	7
Suma et al. 2022 [25]	India	Cross-sectional	25	15	1.14 (0.17)	1.42 (0.15)	Calorimetric	N.A.	N.A.	6
Tasdika et al. 2023 [26]	Bangladesh	Case–control	50	63	1.611 (0.07)	1.644 (0.14)	Atomic absorption	51.0 (0.6)	48.2 (1.5)	6

a includes NPDR. b includes PDR; N.A: Not available.

**Table 2:** Subgroup analysis of magnesium levels in association with diabetic retinopathy.

Sub-Group	Number of Studies	Number of Diabetic Retinopathy	Number of Controls	SMD (95% CI)	I^2^-Index
Continents					
Asia	14	808	789	−1.34 (−1.90; −0.78) *	95.0%
Non-Asian	3	292	343	−0.41 (−0.77; −0.05) *	71.0%
Mg assay methods					
Calorimetric	9	567	560	−1.32 (−1.95; −0.69) *	96.0%
Atomic absorption	5	261	312	−1.23 (−2.23; −0.23) *	94.0%
Enzymatic	3	272	260	−0.69 (−2.27; 0.88)	98.0%
Study Design					
Case–control	10	433	462	−1.14 (−1.86; −0.42) *	94.0%
Cross-sectional	7	667	670	−1.26 (−1.94; −0.57) *	97.0%

* statistical significance; Mg: Magnesium; SMD: standardized mean difference; 95% CI: 95% confidence interval.

**Table 3:** Meta-regression analysis of the magnesium levels and its association with diabetic retinopathy.

Covariates	Estimation Coefficient	Standard Error	*p*-Value	95% CI
NOS	−0.7090	0.3362	0.0350 *	(−214.89; −27.19)
Publication year	0.0618	0.0238	0.009 *	(0.0152; 0.1083)
Sample sizes	−0.0041	0.0021	0.0534	(−0.0082; 0.0001)
Mg assay methods				
Atomic absorption	Reference	Reference		
Calorimetric	−0.0260	0.5505	0.9624	(−1.1050; 1.0531)
Enzymatic	1.2976	0.7399	0.0795	(−0.1525; 2.7478)
Continent				
Asian	Reference	Reference		
Non-Asian	2.3768	0.7396	0.0013 *	(0.9272; 3.8264)

95% CI: 95% confidence interval; NOS: Newcastle–Ottawa Scale. * indicates statistically significant finding; Mg: Magnesium

**Table 4:** GRADE table association of magnesium levels in diabetic retinopathy patients compared with diabetic controls without retinopathy.

Quality Assessment							Summary of Findings	
No. of Patients with Diabetic Retinopathy/Diabetic Without Retinopathy (No. of Studies)	Study Design	Risk of Bias	Inconsistency	Indirectness	Imprecision	Publication Bias	Overall Quality of Evidence	Comment
1100/1132 (17 studies)	Observational studies	Serious ^a^	Very serious ^b^	Not serious	Not serious	Not detected	⨁⨁◯◯ Low	SMD: −1.19; (95% CI = −1.68, −0.70)

GRADE working group grades of evidence. SMD: standardized mean difference. ^a^ Due to the inherent bias of observational studies. ^b^ Owing to the significant heterogeneity levels. ⨁ Indicates upgrading confidence; ◯ indicates downgrading confidence.

## Data Availability

The original contributions presented in this study are included in the article/Supplementary Material. Further inquiries can be directed to the corresponding author.

## Supplementary Materials

The following supporting information can be downloaded at: https://www.mdpi.com/article/10.3390/nu18071162/s1, Table S1. Prisma Checklist. Table S2. Searching strategies for PubMed, Google Scholar, ScienceDirect, and Scopus. Figure S1. Baujat plot for included studies in magnesium levels meta-analysis and diabetic retinopathy [18,20,21,24,25,26,35,36,37,38,39,40,41,42,43,44,45]. Figure S2. Subgroup analysis based on geographical continent for studies pooled in magnesium levels meta-analysis and diabetic retinopathy [18,20,21,24,25,26,35,36,37,38,39,40,41,42,43,44,45]. Figure S3. Subgroup analysis based on study design for studies pooled in magnesium levels meta-analysis and diabetic retinopathy [18,20,21,24,25,26,35,36,37,38,39,40,41,42,43,44,45]. Figure S4. Subgroup analysis based on magnesium assay methods studies pooled in magnesium levels meta-analysis and diabetic retinopathy [18,20,21,24,25,26,35,36,37,38,39,40,41,42,43,44,45].

## Abbreviations

The following abbreviations are used in this manuscript:

IIDM	Insulin independent diabetes mellitus
MeSH	Medical subject heading
Mg	Magnesium
NIDDM	Non-insulin dependent diabetes mellitus
NOS	Newcastle–Ottawa scale
NPDR	Non-proliferative diabetic retinopathy
PDR	Proliferative diabetic retinopathy
PICOS	Population Intervention Comparison Outcome and Study design
PRISMA	Preferred Reporting Items for Systematic Reviews and Meta-Analyses
SD	Standard deviation
SMD	Standardized mean difference
T2DM	Type 2 diabetes mellitus
WHO	The World Health Organization
